# Diagnostic accuracy of telestroke consultation: a Louisiana based tele-network experience

**DOI:** 10.3389/fneur.2023.1141059

**Published:** 2023-06-02

**Authors:** Mugilan Poongkunran, Robin D. Ulep, Gage A. Stuntz, Sara Mitchell, Kenneth J. Gaines, Gabriel Vidal, Daniel Chehebar, Ifeanyi O. Iwuchukwu, Harold McGrade, Alaa E. Mohammed, Richard M. Zweifler

**Affiliations:** ^1^Ochsner Neuroscience Institute, Ochsner Health, New Orleans, LA, United States; ^2^Ochsner Clinical School, New Orleans, LA, United States; ^3^Ochsner Center for Outcomes Research, Office of Epidemiology and Biostatistical Collaborations, Ochsner Clinic Foundation, New Orleans, LA, United States

**Keywords:** telestroke, telemedicine, teleneurology, accuracy, MIMICS

## Abstract

**Background and purpose:**

Telestroke has grown significantly since its implementation. Despite growing utilization, there is a paucity of data regarding the diagnostic accuracy of telestroke to distinguish between stroke and its mimics. We aimed to evaluate diagnostic accuracy of telestroke consultations and explore the characteristics of misdiagnosed patients with a focus on stroke mimics.

**Methods:**

We conducted a retrospective study of all the consultations in our Ochsner Health’s TeleStroke program seen between April 2015 and April 2016. Consultations were classified into one of three diagnostic categories: stroke/transient ischemic attack, mimic, and uncertain. Initial telestroke diagnosis was compared with the final diagnosis post review of all emergency department and hospital data. Sensitivity, specificity, positive predictive value (PPV), negative predictive value (NPV), positive likelihood ratio (LR+) and negative likelihood ratio (LR−) for diagnosis of stroke/TIA versus mimic were calculated. Area under receiver-operating characteristic curve (AUC) analysis to predict true stroke was performed. Bivariate analysis based on the diagnostic categories examined association with sex, age, NIHSS, stroke risk factors, tPA given, bleeding after tPA, symptom onset to last known normal, symptom onset to consult, timing in the day, and consult duration. Logistic regression was performed as indicated by bivariate analysis.

**Results:**

Eight hundred and seventy-four telestroke evaluations were included in our analysis. Accurate diagnosis through teleneurological consultation was seen in 85% of which 532 were strokes (true positives) and 170 were mimics (true negatives). Sensitivity, specificity, PPV, NPV were 97.8, 82.5, 93.7 and 93.4%, respectively. LR+ and LR− were 5.6 and 0.03. AUC (95% CI) was 0.9016 (0.8749–0.9283). Stroke mimics were more common with younger age and female gender and in those with less vascular risk factors. LR revealed OR (95% CI) of misdiagnosis for female gender of 1.9 (1.3–2.9). Lower age and lower NIHSS score were other predictors of misdiagnosis.

**Conclusion:**

We report high diagnostic accuracy of the Ochsner Telestroke Program in discriminating stroke/TIA and stroke mimics, with slight tendency towards over diagnosis of stroke. Female gender, younger age and lower NIHSS score were associated with misdiagnosis.

## Introduction

Stroke is the fifth leading cause of death in the United States, and the leading cause of serious long-term disability (1). Approximately 795,000 people in the United States experience stroke every year and Louisiana is among the top three states in stroke prevalence (5.1%) with reported age-adjusted death rates as high as 88.9 deaths per 100,000 total population (2). Rural communities make up to 25% of Louisiana’s population, with a greater percentage residing outside of areas with accessibility to a primary stroke center, highlighting the need for improved access and quality of healthcare in rural areas (3, 4).

Telemedicine has grown significantly since its implementation in the 1960s and, over the last decade, telestroke has been widely adopted in the care of patients with acute stroke, providing timely access to neurological expertise in rural and other underserved areas (5).

Since the US Food and Drug Administration approval of tissue plasminogen activator (tPA) for acute stroke in 1996, stroke centers have strived to increase the proportion of eligible patients treated with tPA, to improve outcomes for patients presenting within 3 h of symptom onset (6–8). With increasing evidence supporting an improved chain of care enabling rapid triage/diagnosis and increased tPA administration rates among patients presenting to community hospitals, approximately a quarter of US hospitals have implemented telestroke for patients in their emergency departments (9, 10). With expansion of the therapeutic window for patients with large vessel occlusion (LVO) through endovascular thrombectomy (EVT) from 6 to 24 h, there has been an increased focus on triage systems to minimize time to reperfusion and improve outcomes (11, 12).

Despite growing utilization, there is a paucity of data regarding the diagnostic accuracy of telestroke to distinguish between stroke and its mimics as well as specific mimic diagnoses. Due to inherent limitations including lack of physical contact between neurologist and patient, accurate determination of stroke and mimics can, at times, be challenging leading to delayed or missed neurological diagnosis, unnecessary diagnostics and therapeutics and/or wasted resources (13, 14). Hence, we aimed to evaluate diagnostic accuracy of telestroke consultations.

Ochsner Medical Center was the first hospital in Louisiana to implement telestroke and has become one of the fastest growing networks in the country with over 50 active spoke hospitals across Louisiana, Alabama and Mississippi. We performed a retrospective analysis to determine the diagnostic accuracy in patients presenting with symptoms of acute cerebrovascular disease via our large telestroke network. We furthermore aimed to explore the characteristics of misdiagnosed patients with a focus on stroke mimics.

## Methods

Following approval from the Ochsner Institutional Review Board (IRB), we conducted a retrospective observational study of 874 consecutive patients who were evaluated in Ochsner Health’s TeleStroke Program, between April 2015 and April 2016. During the study period, our Telestroke program provided a 24-h per day, 7 days per week, 365 days per year integrated service for recognition and treatment of patients with suspected acute stroke presenting within 12 h of symptom onset. Ochsner Medical Center in New Orleans is a tertiary academic medical center and serves as the “hub,” with vascular neurologists providing collaborative care to “spoke” hospitals via telestroke technology. Telestroke alert is activated by ED physician, or any registered licensed nursing personal at the spoke hospital, if the patient’s presentation suggests acute stroke. Ochsner’s stroke team partners with onsite clinicians/nurses to evaluate, diagnose and directly care for patients. The workflow at the hub includes time-sensitive videoconferencing consultation to the patients at the spoke hospital, mimicking bedside consultation, following American Heart Association/American Stroke Association recommended guidelines for evaluation and management of acute stroke. Initial diagnosis, complete clinical picture, imaging evaluation & recommended treatment was recorded as determined by the telestroke consultant and integrated into patient’s permanent electronic health records, including recommendations on post telestroke care regardless of whether the patient remains at the spoke or requires transfer to a higher level of care.

During the study period, Ochsner Health’s TeleStroke Program had 36 spoke hospitals including Acadian Medical Center – Eunice, LA, Christus St. Frances Cabrini Hospital - Alexandria, LA, Christus St. Patrick Hospital – Lake Charles, LA, Franklin Foundation Hospital – Franklin, LA, Glenwood Regional Medical Center – West Monroe, LA, Ochsner Medical Center – Hancock, MS, Lady of the Sea General Hospital – Cut Off, LA, Mercy Regional Medical Center - Ville Platte, LA, Minden Medical Center – Minden, LA, Natchitoches Regional Medical Center – Natchitoches, LA, Ochsner Baptist Medical Center – New Orleans, LA, Ochsner Medical Center – Baton Rouge, LA, Ochsner Iberville Medical Complex - Plaquemine, LA, Ochsner Health Center – Kenner, LA, Ochsner North Shore Medical Center – Slidell, LA, Ochsner St Anne General Hospital - Raceland, LA, Ochsner Health Center – West Bank, LA, Pointe Coupee General Hospital - New Roads, LA, Ochsner River Parishes Medical Complex - Laplace, LA, Springhill Medical Center – Springhill, LA, St. Charles Parish Hospital – Luling, LA, St. James Parish Hospital – Lutcher, LA, St. Tammany Parish Hospital – Covington, LA, Teche Regional Medical Center - Morgan City, LA, Terrebonne General Medical Center – Houma, LA, and Union General Hospital - Farmerville, LA.

Demographic and clinical data were extracted for each patient, from the Ochsner Telestroke database and the electronic medical record. Specific data elements included: date of birth, gender, race/ethnicity, history of coronary artery disease (CAD), diabetes mellitus (DM), hyperlipidemia (HLD), hypertension (HTN), smoking, prior stroke, medical record number, payor status, date and time of presentation, time last known normal, consultation time, consultation duration (minutes), National Institutes of Health Stroke Scale (NIHSS) score, Glasgow Coma Scale (GCS) score, vascular neurologist name, tPA recommended (yes/no).

Final diagnosis was determined retrospectively via independent review by one of the investigators, which included all emergency department and/or hospital data inclusive of local neurologist (when applicable) final documentation for those admitted. Investigators involved in final diagnosis determination were blinded to the initial consultation diagnosis. Consultations were classified into one of three diagnostic categories: stroke/transient ischemic attack, mimic, and uncertain. The initial telestroke diagnosis was categorized after reviewing all teleconsultant documentation and the most likely diagnostic impression was then assigned. When it was unclear from the record (e.g., only a list of differential diagnoses) “uncertain” was assigned. Stroke was defined as an episode of neurological dysfunction caused by focal cerebral, spinal, or retinal infarction or attributable to a focal collection of blood within the brain parenchyma or ventricular system or attributable to bleeding into the subarachnoid space that is not caused by trauma, or attributable to thrombosis of a cerebral venous structure. Transient ischemic attach (TIA) was defined as brief episodes of neurological dysfunction resulting from focal cerebral ischemia not associated with permanent cerebral infarction. Patients with missing data on initial and final diagnosis, related to any reasons, incomplete investigations or follow-up were excluded from the study.

Initial telestroke diagnosis was compared with the final diagnosis and classified as true positive, true negative, false positive, or false negative for the presence of cerebrovascular disease. Sensitivity, specificity, positive predictive value (PPV), negative predictive value (NPV), positive likelihood ratio (LR+) and negative likelihood ratio (LR-) for diagnosis of stroke/TIA versus mimic were calculated. Area under receiver-operating characteristic curve (AUC) analysis to predict true stroke was performed. Continuous data are described as mean (SD) or median (interquartile range), while categorical data are presented as absolute and relative frequencies (counts and percentages). Bivariate analysis based on the diagnostic categories examined association with sex, age, National Institutes of Health Stroke Scale (NIHSS), stroke risk factors (CAD, DM, HLD, HTN, history of stroke/TIA, smoking), tPA given, bleeding after tPA, symptom onset to last known normal, symptom onset to consult, timing in the day, and consult duration. Logistic regression was performed as indicated by bivariate analysis.

## Results

Of the 904 telestroke consultations conducted between April 2015 and April 2016, 874 patients were included in the study. More than half (67%) had a final diagnosis of cerebrovascular disease, including ischemic stroke (*n* = 460), TIA (*n* = 106) or intracerebral hemorrhage (*n* = 15). Non-cerebrovascular final diagnoses accounted for 240 (27%) of cases with the most common mimics being encephalopathy (*n* = 63), conversion disorder (*n* = 35), migraine (*n* = 34) and seizure (*n* = 33). In fifty-three (6%), the underlying etiology remained uncertain at discharge.

Significant differences were observed between patients with final diagnosis of cerebrovascular disease versus mimic (Table 1). Patients with mimics and uncertain diagnosis were younger, more likely to be female and had lower prevalence of hypertension, diabetes, hyperlipidemia and coronary artery disease than patients with a stroke. A higher proportion of stroke mimics had NIHSS >10 compared to patients with final stroke diagnosis.

**Table 1:** Demographics and clinical characteristics categorized according to the final diagnosis.

Variable	Stroke *N* = 581	Mimic *N* = 240	Uncertain *N* = 53	value of *p*
Age, mean years (SD)	66.6 (14.3)	58.8 (16.5)	58.2 (17.2)	**<0.0001**
Female, *n* (%)	287 (49.4)	150 (62.5)	36 (67.9)	**0.0003**
NIHSS >10, *n* (%)	406 (72.1)	191 (84.5)	49 (94.2)	**<0.0001**
**Comorbidities, *n* (%)**				
Smoking	229 (39.5)	89 (37.4)	23 (44.2)	0.6403
Hypertension	488 (84.0)	170 (70.8)	39 (73.6)	**<0.0001**
Diabetes mellitus	253 (43.6)	80 (33.3)	20 (37.7)	**0.0222**
Hyperlipidemia	295 (50.8)	103 (42.9)	20 (37.7)	**0.038**
Coronary artery disease	160 (27.5)	46 (19.2)	6 (11.3)	**0.0019**
Cerebrovascular disease	165 (28.4)	77 (32.1)	23 (43.3)	0.0672
tPA administered, *n* (%)	190 (32.7)	13 (5.4)	1 (1.9)	**<0.0001**
Bleeding post tPA	23 (12.1)	0 (0.0)	0 (0.0)	0.1757*
**LKN time, no. (%)**				
12:01 AM – 6:00 AM	42 (8.3)	10 (5.5)	1 (2.3)	0.5548*
6:01 AM – 12:00 PM	165 (32.7)	10 (33.9)	17 (39.5)	
12:01 PM – 6:00 PM H	168 (33.3)	10 (33.9)	12 (27.9)	
6:01 PM – 12:00 AM	129 (25.6)	49 (26.8)	13 (30.2)	

Demographics and clinical characteristics categorized according to the final diagnosis. NIHSS, National Institutes of Health Stroke Scale; tPA, tissue plasminogen activator; LKN, last known normal. Bold indicates a significant *p* value. *Fisher’s exact value could not be calculated, therefore Chi-square values were reported.

Overall, accurate diagnosis through teleneurological consultation was seen in 739/874 (85%) of which 532 were strokes (true positives) and 170 were mimics (true negatives). Thirty-seven of these patients remained uncertain of their diagnosis during final evaluation (Table 2). Clinical/demographic differences were observed between misdiagnosed patients and those with accurate diagnosis. Misdiagnosed patients were younger and more likely female. Fifteen (11%) with misdiagnosis received intravenous thrombolysis (IVT) during their teleneurological evaluation, with one experiencing non-fatal bleeding complication.

**Table 2:** Clinical characteristics of patients with accurate diagnosis versus misdiagnosis via teleneurology.

Variable	Accurate diagnosis *N* = 739	Misdiagnosis *N* = 135	value of *p*
Age, mean years (SD)	64.6 (15.19)	60.82 (17.3)	**<0.0103**
Female, *n* (%)	383 (51.8)	90 (66.7)	**0.0013**
NIHSS >10, *n* (%)	174 (24.4)	21 (16.4)	**<0.0414**
**Comorbidities, *n* (%)**			
Smoking	295 (40.1)	46 (34.1)	0.1816
Hypertension	596 (80.1)	105 (77.8)	0.5394
Diabetes mellitus	296 (40.1)	57 (42.2)	0.6375
Hyperlipidemia	356 (48.2)	62 (45.9)	0.6306
Coronary artery disease	182 (24.6)	30 (22.2)	0.5455
Cerebrovascular disease	217 (29.4)	48 (35.6)	0.155
tPA administered, *n* (%)	189 (25.6)	15 (11.1)	**<0.0001**
Bleeding post TPA	22 (11.6)	1 (6.7)	1
Consult duration, mins	17 (9–24)	19 (11–29)	**0.0304**

Clinical characteristics of patients with accurate diagnosis versus misdiagnosis. NIHSS, National Institutes of Health Stroke Scale; tPA, tissue plasminogen activator. Bold indicates a significant *p* value.

Non-cerebrovascular telestroke diagnosis was made in 49/581 (8.4%) of patients with final Stroke/TIA diagnosis (IS, *n* = 36; TIA, *n* = 12; ICH, *n* = 1). The majority of these 37 (75.5%) received an uncertain telestroke diagnosis. Amongst the remaining 12 false negatives cases, the following incorrect diagnoses were made: encephalopathy (*n* = 5), transient global amnesia (*n* = 2), seizure (*n* = 1), conversion disorder (*n* = 1), syncope (*n* = 1), intracranial mass (*n* = 1) and Bell’s palsy (*n* = 1). Twenty-one of the 36 false negative patients presented within 4.5 h of symptom onset and had no known contraindication for IVT, suggesting a percentage of 0.6% of potentially missed thrombolysis due to underdiagnosis.

Non-cerebrovascular final diagnosis was determined in 46/578 (8.0%) who received a diagnosis of cerebrovascular disease via telestroke consultation (i.e., false positive). Etiology remained uncertain in 10; in the remaining, migraine was the most common mimic (*n* = 9) followed by conversion disorder (*n* = 7) and seizure (*n* = 6).

When considering patients with non-uncertain diagnosis, of the 240 patients with final diagnosis of non-cerebrovascular disease, 36 (15%) received a cerebrovascular teleneurological diagnosis (i.e., false positive; IS, *n* = 31; TIA, *n* = 5; Table 3). Eleven (35.5%) false positive patients received IVT, with none experiencing bleeding complication. Of all false positives, 9 had migraine, 7 conversion disorder, 6 seizures and 3 metabolic encephalopathy at final diagnosis. In 34/240 (14.2%), etiology could not be determined during the initial teleneurological consultation.

**Table 3:** Accuracy of initial telestroke diagnosis.

		Final diagnosis		
		Stroke/TIA	Stroke mimic	
Initial diagnosis	Stroke/TIA	True positive 532	False positive 36	PPV = 93.7%
	Stroke mimic	False negative 12	True negative 170	NPV = 93.4%
		Sensitivity = 97.8%	Specificity = 82.5%	

Accuracy of telestroke diagnosis. Patients with uncertain diagnosis during initial consultation or after final evaluation were not included in the analysis.

**Figure 1:**
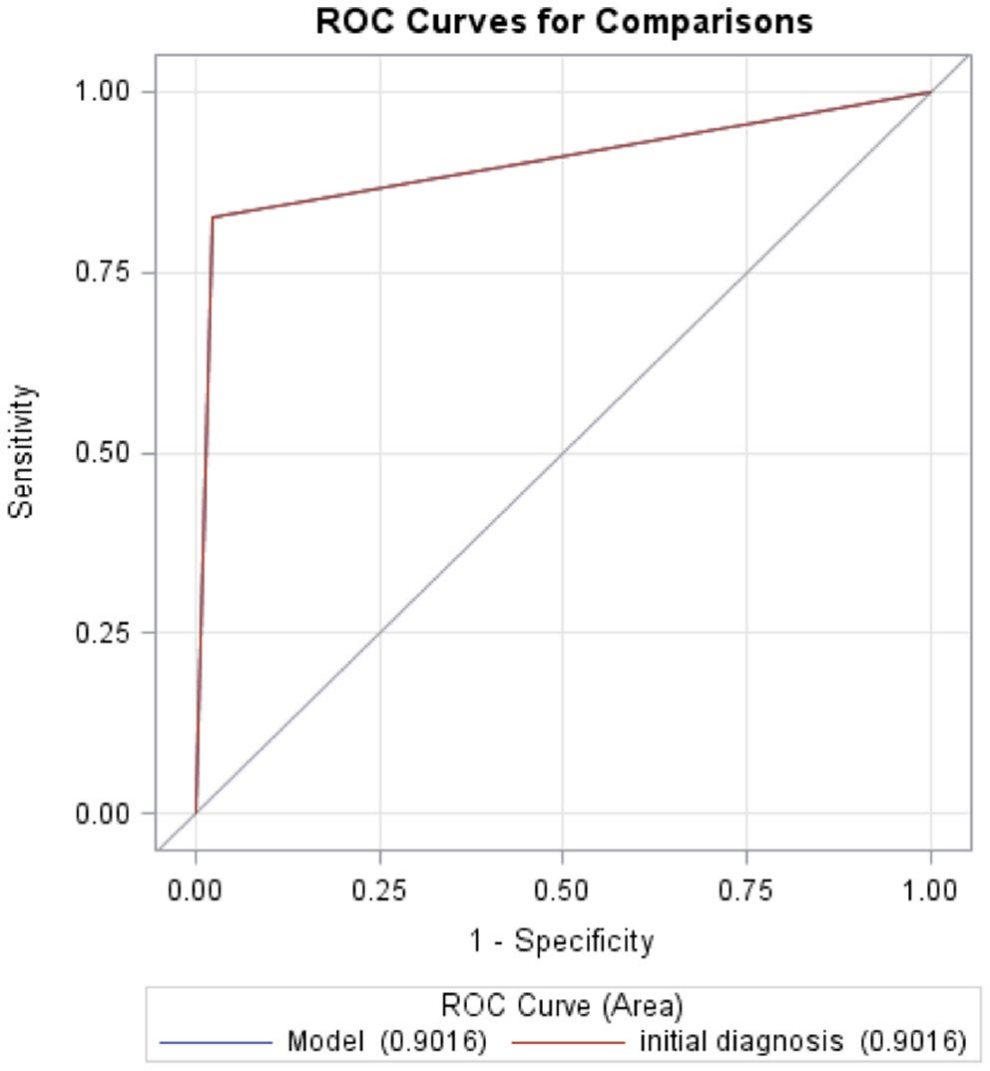
Receiver operating characteristic curves for comparisons. The probability of the test to predict a true stroke or accuracy of the test to discriminate between a true stroke and true mimic is 0.9016.

The sensitivity for diagnosis of cerebrovascular disease was over 97% (sensitivity 97.8%; NPV 93.4%) with a lower tendency towards false-positive diagnosis (specificity 82.5%; PPV 93.7%). Patients with uncertain diagnosis during initial consultations or after final evaluation were not included in this analysis. The positive likelihood ratio and negative likelihood ratio were calculated to be 5.6 and 0.03, respectively. The overall diagnostic accuracy was good with area under the curve (AUC; 95% CI) 0.9016 (0.8749–0.9283; Figure 1).

The odds of misdiagnosis were 1.9 times higher for females than males (Table 4) and the odds of misdiagnosis decreased with increasing age. As regards NIHSS score, the odds of misdiagnosis increased by 1.6 for patients with NIHSS score ≤ 10 compared with NIHSS score > 10. Prolongation of consult time increased the odds of misdiagnosis.

**Table 4:** Logistic regression predicting misdiagnosis.

Variable	OR	CI (95%)
Age	0.987	0.975–0.999
Female	1.915	1.279–2.869
NIHSS <10	1.633	0.970–2.748
Consult duration, mins	1.025	1.008–1.043

## Discussion

We demonstrated a high diagnostic accuracy of the Ochsner Telestroke Program, achieving 85% diagnostic correlation between the teleneurological diagnosis and final discharge diagnosis for patients with both stroke and stroke mimics. The sensitivity for diagnosis of cerebrovascular disease via teleneurology was over 97%, and the overall diagnostic accuracy was very high (AUC 0.9016). Data regarding diagnostic accuracy of teleneurological stroke consultations have been very limited, with reported sensitivity measures ranging wide from 60 to 95% (15–18). Our data comprised of a large cohort of 874 consultations provides greater evidence on the diagnostic accuracy of teleneurological stroke consultations, supporting the utility of telestroke systems in accurate assessment of patients with acute neurological presentations.

In 8.4% of our study population, diagnosis of cerebrovascular disease was missed during telestroke evaluation, with potentially a 0.6% missed thrombolysis or acute intervention due to underdiagnosis. Despite having certified stroke programs, missed opportunities have been reported to be greater than 20% in the ED in both the academic medical centers and community regional referral hospitals (19–21). Consistent with a previous study, we found NIHSS ≤4 and female gender as predictors of stroke chameleons (22). The most common stroke misdiagnosis in our sample was metabolic encephalopathy. Previously reported factors associated with missed stroke diagnosis such as younger age, less vascular risk factors or posterior circulation symptoms could not be established in our study (23, 24). Data regarding stroke chameleons in teleneurology are scarce. Although our volume of underdiagnosed patients was relatively small, our results raise further awareness for missed stroke diagnoses in females and those presenting with lower stroke scale.

As regards overdiagnosis, stroke mimic rates from emergency departments and telestroke networks have been reported from 20 to 45% (25–27)^,^ while the current study found a false positive rate of 27%. Stroke mimics were more common with younger age and female gender and in those with less vascular risk factors. Previous studies also have predominantly showed an inclination towards higher prevalence of mimics in females and younger age groups (28–30). Other reported factors associated with stroke mimics are lower median NIHSS at the time of consult, absence of facial droop, absence of atrial fibrillation and history of seizure disorder. Rapid identification of stroke mimics is critical, especially in those within the thrombolysis treatment window, as administration of thrombolysis to patients with stroke mimics remains prevalent and costly (14). Several stroke mimic prediction models have been formulated to aid in clinical decision-making in both the telestroke and prehospital settings, namely the FABS, simplified FABS, Telestroke Mimic Score (TMS) and Khan Score. These models warrant prospective validation in larger external cohorts (31, 32).

We also investigated the characteristics of patients with overall accurate diagnosis for cerebrovascular or non-cerebrovascular disorders. As for stroke specifically, we found female gender, lower age and lower NIHSS score to be predictors of misdiagnosis. In addition, consult duration was associated with misdiagnosis and may be reflective of the diagnostic challenges in such patients. An understanding of characteristics that place a patient at higher risk of misdiagnosis is valuable to the clinician and may lead to improved accuracy.

Our study has several strengths. The large sample size and inclusion of 36 spokes in the Stroke Belt permitted inclusion of a population with geographic, racial and socioeconomic diversity. Our findings provide insight into the real-world use of teleneurology and contribute to the globally growing experience from different telehealth services. As incomplete documentation was the only criterion for exclusion, discharge documentation was available for all included patients. The stroke neurologist’s clinical diagnosis was the reference standard used in the analyzes and included imaging results, when available. Although diagnostic accuracy is recognized as an important indicator of quality, final diagnoses are not typically documented in teleneurology networks.

Nonetheless, there are inherent limitations in the interpretations of the retrospective study design. Data were not captured discretely in a prospective manner precluding a systematic recording of clinical presentation, risk factors and initial & final diagnostic impression. This can lead to incomplete data capture and inaccuracies in data abstraction. The stroke neurologist’s clinical diagnosis was used as the reference standard to classify patients, with no alternative method to further validate this diagnosis. Additionally, teleconsultation is prone for observer bias, influencing the discharge diagnosis interpretation. Despite the effort to minimize the bias through independent reviewer analysis, diagnostic errors may still persist and the rates of misdiagnosed patients may be underestimated. Because we only analyzed clinical factors previously identified as predictive of stroke mimics and chameleons, we may have missed some important patient characteristics in this data set. Random measurement error and misclassification can lead to dilution bias and underestimation of the effects of the tested risk factors. Finally, the study period preceded guideline-supported thrombectomy which limits the generalizability to telestroke systems using a 24 h window.

## Conclusion

We report high diagnostic accuracy of the Ochsner Telestroke Program in discriminating stroke/TIA and stroke mimics, with slight tendency towards over diagnosis of stroke. Female gender, younger age and lower NIHSS score were associated with misdiagnosis. Future studies should focus on refinement of diagnostic accuracy in these populations.

## Data availability statement

The raw data supporting the conclusions of this article will be made available by the authors, without undue reservation.

## Ethics statement

The studies involving human participants were reviewed and approved by Ochsner Health. Written informed consent for participation was not required for this study in accordance with the national legislation and the institutional requirements.

## Author contributions

RZ conceived the study and contributed to the study design. GV, DC, II, and HM contributed to acquisition of data. GS and SM contributed to study design and data aquisition. AM performed the statistical analysis. MP was involved in data analysis, data interpretation and drafting of the manuscript. RZ provided critical revision of the manuscript and important intellectual contribution in data analysis and interpretation. All authors contributed to the article and approved the submitted version.

## Conflict of interest

The authors declare that the research was conducted in the absence of any commercial or financial relationships that could be construed as a potential conflict of interest.

## Publisher’s note

All claims expressed in this article are solely those of the authors and do not necessarily represent those of their affiliated organizations, or those of the publisher, the editors and the reviewers. Any product that may be evaluated in this article, or claim that may be made by its manufacturer, is not guaranteed or endorsed by the publisher.
